# Rates, indications, and outcomes of caesarean section deliveries: A comparison of tribal and non-tribal women in Gujarat, India

**DOI:** 10.1371/journal.pone.0189260

**Published:** 2017-12-27

**Authors:** Gayatri Desai, Ankit Anand, Dhiren Modi, Shobha Shah, Kalpana Shah, Ajay Shah, Shrey Desai, Pankaj Shah

**Affiliations:** 1 Kasturba Maternity Hospital (KMH), SEWA Rural, Bharuch, Gujarat, India; 2 Community Health Project (CHP), SEWA Rural, Bharuch, Gujarat, India; 3 Women’s Health and Training Center, SEWA Rural, Bharuch, Gujarat, India; Liverpool School of Tropical Medicine, UNITED KINGDOM

## Abstract

**Background:**

Even though the caesarean section is an essential component of comprehensive obstetric and newborn care for reducing maternal and neonatal mortality, there is a lack of data regarding caesarean section rates, its determinants and health outcomes among tribal communities in India.

**Objective:**

The aim of this study is to estimate and compare rates, determinants, indications and outcomes of caesarean section. The article provides an assessment on how the inequitable utilization can be addressed in a community-based hospital in tribal areas of Gujarat, India.

**Method:**

Prospectively collected data of deliveries (N = 19923) from April 2010 to March 2016 in Kasturba Maternity Hospital was used. The odds ratio of caesarean section was estimated for tribal and non-tribal women. Decomposition analysis was done to decompose the differences in the caesarean section rates between tribal and non-tribal women.

**Results:**

The caesarean section rate was significantly lower among tribal compared to the non-tribal women (9.4% vs 15.6%, p-value < 0.01) respectively. The 60% of the differences in the rates of caesarean section between tribal and non-tribal women were unexplained. Within the explained variation, the previous caesarean accounted for 96% (p-value < 0.01) of the variation. Age of the mother, parity, previous caesarean and distance from the hospital were some of the important determinants of caesarean section rates. The most common indications of caesarean section were foetal distress (31.2%), previous caesarean section (23.9%), breech (16%) and prolonged labour (11.2%). There was no difference in case fatality rate (1.3% vs 1.4%, p-value = 0.90) and incidence of birth asphyxia (0.3% vs 0.6%, p-value = 0.26) comparing the tribal and non-tribal women.

**Conclusion:**

Similar to the prior evidences, we found higher caesarean rates among non-tribal compare to tribal women. However, the adverse outcomes were similar between tribal and non-tribal women for caesarean section deliveries.

## Introduction

The maternal and neonatal health outcomes among tribal and indigenous communities are worse compared to non-tribal populations in low-middle income countries [1–2]. The availability of comprehensive emergency obstetric and neonatal care (CEmONC) is inequitable in low resource settings such as remote rural and tribal areas [3–5]. Hence, providing CEmONC can improve maternal and neonatal health in tribal regions [4]. One of the essential components of CEmONC is the delivery of a foetus through a caesarean section. Caesarean section, when indicated, can reduce maternal and neonatal mortality and morbidity [5–7]. Some of the common, life-threatening indications for a caesarean section are obstructed labour, selected breech delivery, mal-presentation of the foetus and foetal distress [7]. Investing in the caesarean section is also found to be cost-effective [8]. The World Health Organization (WHO) earlier recommended around 5–15% rate of caesarean section in any population [6,9]. However, WHO recently suggested that they do not recommend a specific rate at either a country-level or a hospital-level [10]. Tribal population accounts for 8.4% (104 million) of the total population of India [11]. The overall rate of caesarean section delivery in 2015–16 is around 17.2% in India, increased from 8.5% in 2005–06 [12,13]. However, the caesarean section rate is estimated to be low in rural areas (12.9%) [12]. Lack of availability of emergency obstetric services, knowledge and financial constraint are some of the important factors for low caesarean section rate among rural tribal women [14–16]. In recent times, the incidences of caesarean section are on a rise in India [12, 17–19]. It would be important to study whether the increase in trend of caesarean section is equitable comparing the tribal to non-tribal communities. Additionally, the results of implementing the practices to limit the increasing rates of caesarean needs to be described.

The clinical indications of caesarean section among rural tribal women have not been studied in India. In general, there is a lack of data on indications and outcomes of caesarean section among tribal communities [6–7]. As the institutional deliveries and caesarean section are increasing, there is a need for better understanding of indication and outcomes of caesarean section [19]. The information on the indications of the caesarean section will be useful to clinicians, and public health practitioners in improving maternal health outcomes in tribal regions in India and countries with similar needs [7, 19]. Other condition such as sickle cell disease found predominantly among tribal women is also a risk for adverse pregnancy outcomes [20]. It will be crucial to compare the outcomes between tribal and non-tribal women with caesarean and vaginal deliveries. There are studies reported negative or no influences of caesarean on perinatal mortality in low-middle income countries where the caesarean rates are high [21–22]. There are also possible benefits of caesarean in settings where the caesarean rates are very low, due to unavailability of caesarean when needed [21–23]. This study reports the analyses of maternal admissions in the community-based hospital of SEWA-Rural (Kasturba Maternity Hospital) providing tribal-friendly CEmONC services in the Jhagadia block of Bharuch district, Gujarat. The objective of this study is to estimate rates, indications, determinants, and outcomes of caesarean section comparing tribal and non-tribal women in the context of a tribal friendly hospital providing CEmONC.

## Data and methods

We used the data of all women who delivered at the Kasturba Maternity Hospital (KMH), a hospital managed by SEWA Rural, a voluntary, not-for-profit organization. KMH has been providing maternal and neonatal health services in this area since 1980 along with community-based services in surrounding areas [24]. The hospital functions in Jhagadia block of Bharuch in the western Indian state of Gujarat. The total population of Jhagadia block is around 185,000, of which 70% is tribal [11]. The hospital provides free clinical services to pregnant women and newborns. The hospital works as a first referral unit and is the largest provider of maternal health care in the Bharuch district and nearby areas.

The study is based on secondary analyses of data which was primarily collected for delivery and monitoring of services at the hospital. The data is part of the hospital program to provide quality health services to the remote and tribal areas of Gujarat. The ethical approval for the use of this data has been obtained from SEWA Rural Institutional Ethics Committee (IEC). The SEWA Rural IEC reviewed this data and allowed its use for analyses and publication. The IEC have also waived the need for the informed consent of the patient, given that the anonymity of the patient will be maintained. Any personal information of the patient was removed from the datasets before analysis.

### Study setting

We used the data of all women who delivered at the Kasturba Maternity Hospital (KMH), a hospital managed by SEWA Rural, a voluntary, not-for-profit organization. KMH has been providing maternal and neonatal health services in this area since 1980 along with community-based services in surrounding areas [24]. The hospital functions in Jhagadia block of Bharuch in the western Indian state of Gujarat. The total population of Jhagadia block is around 185,000, of which 70% is tribal [11]. The hospital provides free clinical services to pregnant women and newborns. The hospital works as a first referral unit and is the largest provider of maternal health care in the Bharuch district and nearby areas.

The tribal population native to the study area is known as Vasava, and belongs to the Bhil tribe. As with other tribal communities, Vasava tribe has its own language and customs which are different from the mainstream culture; though this is changing as they are increasingly connected with the mainstream culture. Agriculture is the primary occupation with a large percentage of the population working as landless labourers [25].

KMH is a 200-bed tribal-friendly first referral unit providing CEmONC care to surrounding villages. The CEmONC services at the hospital include services from full-time clinicians including obstetricians, anaesthetists and internists. Along with the clinician, the hospital has a functional operation theatre, blood storage centre, ultrasound, and inpatient facility [24]. The mission of the hospital is to serve the most underprivileged tribal patients. Therefore, it is an empanelled provider for the government sponsored health insurance schemes to facilitate almost free or highly subsidized services to the poor.

### Data sources and sample size

**Fig 1:**
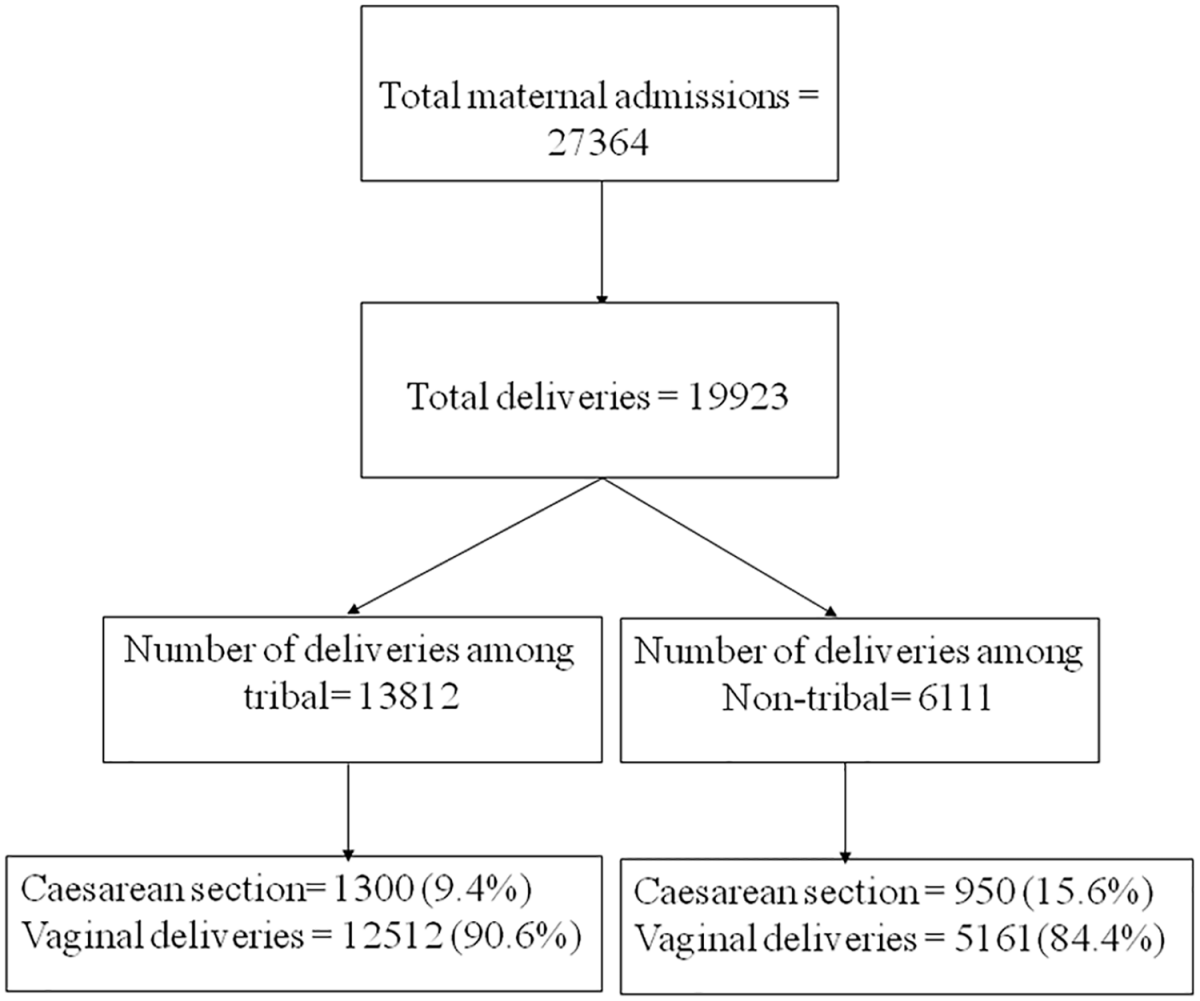
Number of deliveries and caesarean sections in KMH (2010–16), Bharuch, Gujarat.

The hospital maintains a prospective registry for all admissions that is maintained by care providers and subsequently digitized by trained staff. A team of care providers including gynaecologists ensured the accuracy of the entire dataset, including that of indications and outcomes of all deliveries. Women who delivered from April 2010 to March 2016 were included in the study. The definition of tribal is as per the specifications from the government of India [26]. The description of admission and outcomes is shown in Fig 1.

### Independent and dependent variables

The deliveries were categorized as caesarean section and vaginal deliveries, and are the primary dependent variables. The potential determinants of caesarean section were maternal age, parity, maternal education, gestational week, haemoglobin status, government scheme, distance from the health facility and child’s gender. The adverse outcomes of deliveries examined for this analyses were still birth, low birth weight (less than 5.5 lbs/2500 grams), survival status of the neonate at the time of discharge from the hospital (case fatality rate) and birth asphyxia (no cry after delivery).

### Statistical analyses

Rate, indication and outcomes of caesarean section were represented by percentages and counts comparing tribal and non-tribal women. Still birth rate and neonatal death rate are expressed per 1000 total (live + still) deliveries and per 1000 live births respectively. Logistic regression was used to estimate the odds ratio caesarean section by background characteristics as potential determinants. The odds ratio was adjusted for Mother’s age, Mother’s education, Parity, Mother’s haemoglobin, Gestational week, Child’s gender, Number of ANC visits by mother and Distance from the health facility. The odds ratio of caesarean section was estimated for tribal and non-tribal women. Decomposition analysis was done to decompose the differences in the rates of caesarean section between tribal and non-tribal women. Fairline modification of Blinder-Oaxaca decomposition for the logit models was used [27]. Various indications for caesarean section among tribal women were compared with non-tribal women. The odds ratio of each pregnancy outcome of caesarean section and vaginal deliveries was calculated. The odds ratios were separately calculated for caesarean section and vaginal deliveries comparing tribal and non-tribal women. All of the odds ratios were reported with 95% confidence interval. Strobe guidelines were followed for reporting the results. Microsoft Excel 2007 was used to compile the data and STATA Version 12.0 was used for statistical analyses [28].

## Results

The total number of deliveries were 19923 (tribal = 13812, non-tribal = 6111). The number of vaginal deliveries and those via caesarean section by ethnicity are given in Fig 1. Socioeconomic and other important characteristics of women delivered in the hospital are given in Table 1. Educational attainment was higher among non tribal women. Anaemia was higher among tribal women compared to non tribal women. A higher percentage of non-tribal women were coming from areas more than 100 kilometres away from the hospital compared to tribal women. Still births, low birth weight and no cry at birth were significantly higher among tribal women compared to non-tribal women.

**Table 1:** Socioeconomic and clinical characteristics of tribal and non-tribal women in KMH (2010–16), Bharuch, Gujarat.

		Tribal (N = 13812 deliveries)		Non Tribal (N = 6111 deliveries)		p-value
		N	%	N	%	
**Mother’s age**	**15–19**	672	4.9	194	3.2	0.000
	**20–24**	9,992	72.4	3,735	61.1	
	**25–29**	2,511	18.2	1,666	27.3	
	**30 and above**	635	4.6	514	8.4	
**Mother’s education**	**No education**	2,584	18.8	867	14.2	0.000
	**1–7 years**	5,795	42.1	2,144	35.2	
	**8–12 years**	5,059	36.8	2,707	44.4	
	**12 years and more**	315	2.3	378	6.2	
**Parity**	**0**	7,411	53.7	3,184	52.1	0.020
	**1**	4,166	30.2	1,950	31.9	
	**2**	1,669	12.1	681	11.1	
	**3 and above**	566	4.1	296	4.8	
**Mother’s haemoglobin at delivery**	**<7.0**	660	4.8	159	2.6	0.000
	**7.1–10.0**	5,275	38.5	1,883	31.1	
	**10.1–11.0**	3,884	28.3	1,558	25.7	
	**>11.0**	3,896	28.4	2,464	40.6	
**Previous caesarean**	**No**	13,404	97.9	5,750	94.1	0.000
	**Yes**	408	2.1	361	5.9	
**Gestational week**	**<36 weeks**	2,439	17.7	749	12.3	0.000
	**37–42 weeks**	11,362	82.3	5,358	87.7	
	**>42 weeks**	11	0.1	4	0.1	
**Child’s gender**	**Female**	6,560	47.5	2,945	48.2	0.364
	**Male**	7,252	52.5	3,166	51.8	
**Number of ANC visits**	**0**	1,064	7.7	474	7.8	0.219
	**1–2**	6,810	49.4	2,936	48.1	
	**3 and above**	5,903	42.8	2,691	44.1	
**Distance from the health facility (in Kilometres)**	**0–25**	11,102	80.4	3,766	61.6	0.000
	**26–50**	1,391	10.1	406	6.6	
	**51–100**	955	6.9	973	15.9	
	**>100**	364	2.6	966	15.8	
**Still Birth**	**No**	13,358	96.7	6,003	98.2	0.000
	**Yes**	450	3.3	107	1.8	
**Birth weight <2500 grams**	**No**	7,575	56.7	4,083	68.0	0.000
	**Yes**	5,780	43.3	1,919	32.0	
**Immediate cry at birth**	**No**	406	3.0	146	2.4	0.019
	**Yes**	12,951	97.0	5,857	97.6	
**Neonatal deaths at hospital**	**No**	13,341	99.9	5,990	99.8	0.114
	**Yes**	17	0.1	13	0.2	

### Rates and determinants of caesarean rates among tribal and non tribal women

**Fig 2:**
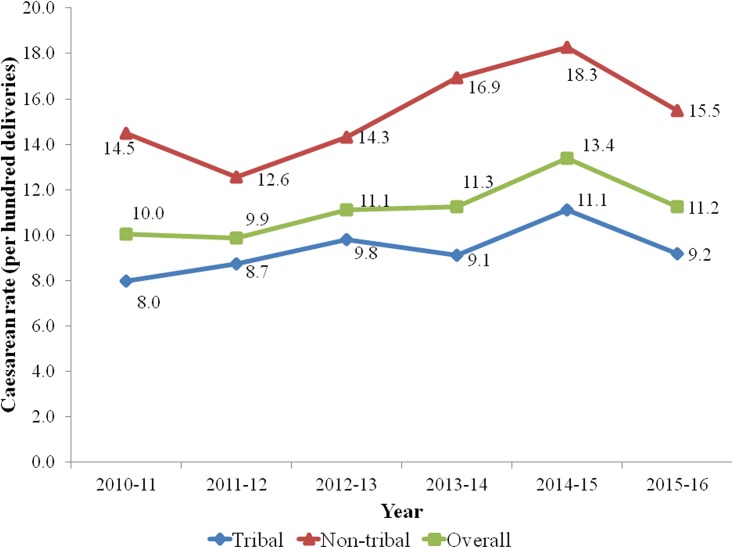
Annual caesarean rates (%) among tribal and non-tribal women in KMH (2010–16), Bharuch, Gujarat.

The caesarean section rates for tribal and non-tribal women were 9.4% (n = 1300) and 15.6% (n = 950) respectively (Fig 1). The gaps in caesarean section rates between tribal and non-tribal women were similar in the six year study timeframe (Fig 2). Our findings demonstrate an increasing trend of caesarean section rates in both tribal and non-tribal women. Overall, 14,539 (73%) out of total deliveries received the benefit of various government sponsored health insurance schemes and did not bear any out of pocket expenditure for delivery.

Increasing age of the mother was related to increasing caesarean section rates in tribal women (Table 2). Education was significantly associated with increasing caesarean among non tribal women. The odds ratio was significantly lower among women with parity 1 and above compared to women with zero parity in tribal and non tribal women alike. Gender of the child, gestational weeks and mother’s haemoglobin count were not significantly associated with caesarean section. The caesarean rate among women who had caesarean in the past was very high compared to women who did not have any previous caesarean. Tribal Women coming from areas in the range of 25–50 kilometres from the hospital have higher caesarean section rate compared to women coming from the areas less than 25 kilometres.

**Table 2:** Numbers and rates of caesarean section by selected determinants among tribal and non-tribal women.

		Tribal (N = 1300 caesarean deliveries)			Non tribal(N = 950 caesarean deliveries)		
		Number (Caesarean Rate*)	Adjusted odds ratio	P-value	Number (Caesarean Rate*)	Adjusted odds ratio	P-value
	**Overall**	1300(9.4)			950(15.5)		
**Mother’s age**	**15–19**	45(6.7)			23(11.9)		
	**20–24**	880(8.8)	1.39(1.01–1.90)	0.043	547(14.6)	1.26(0.80–1.98)	0.323
	**25–29**	277(11.0)	1.99(1.40–2.82)	0.000	276(16.6)	1.34(0.83–2.18)	0.234
	**30 and above**	98(15.4)	3.32(2.19–5.03)	0.000	103(20.0)	1.69(0.98–2.94)	0.061
**Mother’s education**	**No education**	222(8.6)			91(10.5)		
	**1–7 years**	516(8.9)	1.11(0.96–1.28)	0.146	327(15.3)	0.97(0.81–1.16)	0.746
	**8–12 years**	509(10.1)	1.10(0.76–1.60)	0.602	435(16.1)	1.45(1.07–1.98)	0.018
	**12 years and more**	46(14.6)	1.01(0.84–1.22)	0.875	94(24.9)	0.73(0.54–0.98)	0.039
**Parity**	**0**	728(9.8)			493(15.5)		
	**1**	424(10.2)	0.43(0.36–0.51)	0.000	332(17.0)	0.40(0.32–0.50)	0.000
	**2**	107(6.4)	0.31(0.24–0.40)	0.000	95(14.0)	0.37(0.26–0.52)	0.000
	**3 and above**	41(7.2)	0.39(0.27–0.57)	0.000	30(10.1)	0.42(0.26–0.70)	0.001
**Mother’s haemoglobin at delivery**	**<7.0**	61(9.2)			17(10.7)		
	**7.1–10.0**	515(9.8)	0.91(0.67–1.23)	0.525	277(14.7)	0.91(0.51–1.61)	0.735
	**10.1–11.0**	337(8.7)	0.74(0.54–1.01)	0.060	207(13.3)	0.74(0.42–1.33)	0.321
	**>11.0**	382(9.8)	0.84(0.61–1.15)	0.273	441(17.9)	1.01(0.57–1.79)	0.969
**Gestational week**	**<36 weeks**	199(8.2)			89(11.9)		
	**37–42 weeks**	1099(9.7)	1.17(0.98–1.39)	0.078	859(16.0)	1.14(0.88–1.49)	0.325
	**>42 weeks**	2(18.2)	1.94(0.32–11.89)	0.473	2(50.0)	7.98(0.97–65.74)	0.054
**Previous caesarean**	**No**	1016 (7.6)			657 (11.4)		
	**Yes**	284 (69.6)	44.7(34.74–57.51)	0.000	293 (81.3)	57.51(41.97–78.82)	0.000
**Child’s gender**	**Female**	599(9.1)			429(14.6)		
	**Male**	701(9.7)	1.10(0.97–1.24)	0.144	521(16.5)	1.16(0.98–1.36)	0.076
**Number of ANC visits**	**0**	117(11.0)			45(9.5)		
	**1–2**	520(7.6)	0.60(0.48–0.76)	0.000	363(12.4)	1.01(0.70–1.46)	0.937
	**3 and above**	663(11.2)	0.91(0.72–1.16)	0.465	542(20.1)	1.67(1.16–2.41)	0.006
**Distance from the health facility (in Kilometres)**	**0–25**	975(8.8)			577(15.3)		
	**26–50**	172(12.4)	1.31(1.07–1.59)	0.007	71(17.5)	1.20(0.88–1.65)	0.248
	**51–100**	112(11.7)	1.25(1.00–1.58)	0.055	169(17.4)	1.28(1.03–1.59)	0.026
	**>100**	41(11.3)	1.07(0.74–1.56)	0.704	133(13.8)	1.11(0.88–1.40)	0.393

*per 100 deliveries

### Decomposition of caesarean section rates between tribal and non-tribal women

Decomposition analysis decomposed differences found between tribal and non-tribal women to other selected variables (Table 3). Overall 40% of variation was explained by the variables. Maternal age was responsible for about 15.4% of explained variation. Previous caesarean section was accounted for 96% of explained variation between tribal and non-tribal women. Parity also significantly explained the difference between tribal and non-tribal women. Distance from health facility accounted for 18% of explained variation, though it was non-significant.

**Table 3:** Results of decomposition analysis on caesarean rate between tribal and non-tribal women.

	Absolute difference	Percentage contribution to total differences	Percentage contribution to explained differences	p-value
**Overall difference in caesarean rates between tribal and non-tribal**	0.041	100.0		
**Unexplained difference in caesarean rates between tribal and non-tribal**	0.025	60.0		
**Explained difference in caesarean rates between tribal and non-tribal**	0.017	40.0	100.0	
**Mother’s age**	0.003	7.2	15.4	0.058
**Parity**	-0.006	-14.4	-37.7	0.000
**Previous caesarean**	0.016	38.4	96.0	0.000
**Mother’s education**	0.000	0.0	-1.0	0.335
**Haemoglobin status**	0.000	0.0	2.4	0.725
**Number of ANC visits**	0.000	0.0	1.9	0.338
**Distance from the health facility**	0.003	7.2	18.1	0.143
**Gender**	0.000	0.0	0.3	0.595
**Year**	0.001	2.4	4.1	0.061

### Clinical indication of caesarean section

The most common indications for caesarean section were foetal distress followed by previous caesarean section and breech presentation (Table 4). Among non-tribal women, the most common indicators were the previous caesarean section, followed by foetal distress, breech and prolonged labour. Previous caesarean sections were significantly higher among non tribal women. Similarly, transverse lies were higher in tribal compared to non-tribal women. Pregnancy induced hypertension being the most common secondary indications for caesarean section in both tribal and non-tribal women.

**Table 4:** Primary and secondary indications for caesarean section.

	Tribal (N = 1300 Caesarean sections)		Non-tribal (N = 950 Caesarean sections)		P-value
	Number	%	Number	%	
**Primary indication**					
Foetal distress	406	31.2	291	30.6	0.761
Previous Caesarean sections	311	23.9	315	33.2	0.000
Prolonged Labour	145	11.2	115	12.1	0.486
Breech	208	16.0	116	12.2	0.011
Transverse lie	67	5.2	27	2.8	0.007
Obstructed Labour	32	2.5	16	1.7	0.208
Placenta previa	35	2.7	15	1.6	0.077
Multiple births	26	2.0	12	1.3	0.180
Cephalopelvic disproportion	24	1.8	11	1.2	0.193
Placental abruption	17	1.3	11	1.2	0.752
Failed Medical induction of labour	9	0.7	7	0.7	0.901
Others	100	7.7	80	8.4	0.582
**Secondary indication**					
Pregnancy induced hypertension	205	15.8	146	15.4	0.796
Eclampsia	62	4.8	32	3.4	0.101
Sickle Cell disease	37	2.8	0	0.0	
Anaemia	75	5.8	39	4.1	0.075
Oligohydramnios	20	1.5	14	1.5	0.901

### Outcomes of caesarean section deliveries

There was no difference in the case fatality rate among neonates comparing the tribal and non-tribal in caesarean section deliveries (Table 5). Percentage of no cry after birth was similar among tribal women in both caesarean section and vaginal deliveries. The percentage of the neonates who weighed less than 2.5 kilograms was also similar in caesarean section and vaginal deliveries. Percentage of neonates who weighed less than 2.5 kilograms was higher among tribal compared to non-tribal women. Still birth rate was lower in caesarean section deliveries. However, the difference between tribal and non-tribal was significant in both types of deliveries. Around 90% of women who had still birth had no foetal heart sound on admission suggesting the foetus had already died before the women arrived at the KMH hospital.

**Table 5:** Adverse clinical outcomes after caesarean section and vaginal deliveries among tribal and non-tribal women.

	Caesarean section (N = 2250)			Vaginal deliveries (N = 17673)		
	Tribal	Non-tribal	Unadjusted P-value	Tribal	Non-tribal	Unadjusted P-value
**Birth Asphyxia (no cry after Birth) n(%)**	4(0.3)	6(0.6)	0.267	56(0.5)	20(0.4)	0.541
**Birth weight <2500 grams n(%)**	572(44.0)	283(29.8)	0.000	5595(44.7)	1713(33.2)	0.000
**Still birth rate n(per 1000 live + still births)**	35(26.9)	8(8.4)	0.002	415(33.2)	99(19.2)	0.000
**Case fatality rate n(in hospital neonatal deaths per 1000 live births)**	17(13.1)	13(13.7)	0.901	263(19.0)	84(13.7)	0.010

## Discussion

This is one of the few studies to compare the rates, indications and outcomes of caesarean section between tribal and non-tribal women in India. Studies in the past have shown lower caesarean section rate in India compare to other Asian countries [29–31]. Studies have also reported differences in caesarean section rates and other maternal health related indicators between tribal and non-tribal [1,4,32–33]. Higher caesarean rates have been reported among non-tribal women compared to tribal women in other studies in India [12,32–33]. Delaying the childbearing and improving the care-seeking behaviour among the tribal women in our study population might help to reduce the difference in the caesarean section rate [33–34]. Antenatal care visits during pregnancy may lead to the identification of complication and consequently higher caesarean section rates among women who have 3 or 4 ANC visits [34–35]. Age, education and parity of the women have been reported as important determinants of caesarean section in many studies as similar to our findings [19,30,34–35]. Our data could only explain around 40% of differences in the caesarean rates between tribal and non tribal. More research may be required for exploring the gap between tribal and non-tribal women. We also found higher risk of caesarean among women with previous caesarean as also depicted in many studies [7,36–37]. Household surveys also suggest availability, accessibility and affordability are one of the factors for less use of emergency obstetrics services [15,32,34,38]. In our study, the hospital provides round the clock, nearly free of cost, and good quality services, thereby facilitate availability and affordability. In addition, emergency ambulance service also functions at zero cost to the patient and makes the hospital easily accessible. This may also have contributed to the higher caesarean section rates among tribal women in our study compared to other studies.

Previous caesarean section, foetal distress and mal-presentation of the foetus (Breech and Transverse Lie) were the most common clinical indication for caesarean section in our study. Our results are relatively consistent with other studies in India and other countries [29,37]. Similar to our findings, the previous caesarean section, foetal distress and mal-presentation of the foetus (Breech and Transverse Lie) were reported as major indication of caesarean section in many studies [7,29,37]. Obstructed labour was also reported as an important indication in few studies [7]. However, obstructed labour was not a major indication of caesarean in our study. There are also variations between studies for reporting primary indications for caesarean section [27,39]. There is a need for universal clinical indications in which caesarean section can be performed [39]. We did not find any differences in neonatal outcomes comparing caesarean and vaginal deliveries. Studies have suggested non significant influences on neonatal morbidity and mortality in setting where the caesarean rates are very high (21–23). However, as suggested by WHO, caesarean section can be a life saving tool if performed in optimal condition and indications [10]. However, it might be noted that most of the women (70%) who had still birth were admitted with no foetal heart rate at the time of admission. The intra uterine foetal death before arriving to the hospital might have resulted in higher still birth rate in vaginal delivery. Considering the almost lack of data about caesarean section in tribal communities in India, it is critical to collect, analyze and review data about caesarean section rates segregated by caste [39].

There are a few lessons learned regarding how to develop tribal friendly health care facility for reducing the inequity of the health services in the tribal area. Some of the government sponsored policies that enabled the KMH to develop tribal friendly hospital, such as the grant-in-aid scheme and Chiranjivi Scheme could be explored for replicating in other tribal predominant states in India [40–41]. At the same time, it is important to take note of the KMH team’s efforts to prevent unnecessary caesarean section among tribal and non-tribal women by creating the culture of rational practice, and monitoring of caesarean section rates on the ongoing basis. The most common barrier for availing the caesarean section in tribal areas is the lack of qualified human resources such as obstetricians and anaesthesiologists [3,6,33–34]. Although the government is working towards improving human resources in the tribal area, more needs to be done to ensure every woman in need of life saving surgery of caesarean Section has access to it so that the Development Goals related to the maternal health can be achieved [40,42].

There are some limitations of this study. This study was done in the context of a tribal friendly hospital. The findings may not be generalized to other areas of India. Most of the women (90%) who had still birth were admitted with no foetal heart rate, which may cause higher still birth rate in vaginal delivery. It would have been useful to compare other outcomes of the caesarean section such as APGAR (Appearance, Pulse, Grimace, Activity and Respiration) score along with the intra-operative and post-operative complications. Other mother related complication and delays in receiving services were also not available. The information was not available for the analysis. The study is based on maternal admissions; some women may have more than one delivery during the period of study.

## Conclusion

It is one of the few studies which have compared indications and outcomes of caesarean section among tribal and non-tribal women in India. The caesarean rates were higher among non-tribal compared to tribal women. However, the adverse outcomes are similar among the tribal women compared to the non-tribal women who underwent the caesarean section. The previous caesarean section, maternal age, maternal education, parity and number of ANC visits are some of the important determinants of caesarean rates. The previous caesarean section among non-tribal and foetal distress in tribal was the most common indication of caesarean. Further research investigating and addressing the factors identified in this study are recommended.

## Supporting information

S1 File The dataset of all deliveries in KMH (2010–16), Bharuch, Gujarat. (XLSX)

## Abbreviations

ANC: Antenatal Care
CEmONC: Comprehensive Emergency Obstetric and Neonatal Care
KMH: Kasturba Medical Hospital
SEWA-Rural: Society for Education, Welfare and Action-Rural
WHO: World Health Organization
